# Exploring older adults’ views about alcohol consumption: implications for brief interventions with older heavy drinkers

**DOI:** 10.1186/1940-0640-8-S1-A86

**Published:** 2013-09-04

**Authors:** Graeme B Wilson, Eileen FS Kaner, Jonathan Ling, Ann Crosland, Karen McCabe, Catherine A Haighton

**Affiliations:** 1 Institute of Health and Society, Newcastle University, Newcastle upon Tyne, Tyne and Wear, UK; 2 Department of Pharmacy, Health & Wellbeing, Sunderland University, Tyne and Wear, UK

## 

Increasing alcohol consumption among older individuals in the UK is a public health concern. Although brief interventions (BIs) can effectively reduce heavy drinking in older age groups generally, social and contextual factors may influence implementation and effectiveness. Identities offer a useful theoretical concept to explain why the potential for public health messages to reduce rates of heavy drinking in this sector has not been realised, and can inform preventive approaches. A qualitative study explored older people’s reasoning about drinking in later life and how this interacted with health concerns, to inform future, targeted prevention in this group. A diverse sample of older adults in North East England (ages 51-90) participated in interviews (n=24, 12m, 12f) and three focus groups (participants n=27). Data were analysed using grounded theory and discursive psychology methods. Older adults portrayed drinking less alcohol as an appropriate response if one experienced impaired health. However continued heavy drinking could be presented as normal behaviour for someone experiencing relative wellbeing in later life, or if ill health was construed as unrelated to alcohol consumption. When talking about alcohol use older people oriented strongly towards opposed identities of normal or problematic drinker, defined by propriety rather than health considerations. These identities were flexible in older people’s talk since they could be applied to either moderate or heavy drinking. Older people displayed scepticism about health advice on alcohol when avoiding stigmatised identity as a drinker. Findings indicate that older UK adults do not recognise unhealthy drinking as distinct from dependent drinking, and are highly sensitive to stigma when discussing alcohol consumption. Preventive strategies should encourage older people’s identification of heavy drinking as neither healthy nor synonymous with dependence. BIs for heavy drinkers in later life should be tailored to address lay reasoning that is resistant to recognition of health risks.

